# 3D-Printed Sievenpiper Metasurface Using Conductive Filaments

**DOI:** 10.3390/ma13112614

**Published:** 2020-06-08

**Authors:** Pablo Stuardo, Francisco Pizarro, Eva Rajo-Iglesias

**Affiliations:** 1 Escuela de Ingeniería Eléctrica, Pontificia Universidad Católica de Valparaíso, Valparaíso 2362804, Chile; pablo.stuardo.o@mail.pucv.cl; 2 Department of Signal Theory and Communications, University Carlos III of Madrid, 28911 Leganés, Spain; eva.rajo@uc3m.es

**Keywords:** electromagnetic metasurfaces, 3D-printing, conductive filaments, stop-band

## Abstract

This article presents the design, construction and measurement of different 3D-printed Sievenpiper metasurfaces. The structures were printed using a conductive filament combined with regular polylactic acid PLA. Measurement shows a good agreement on the electromagnetic behaviour of the stop-bands generated by the fully 3D-printed metasurface and the simulated ideal cases, but with higher transmission losses due to the characteristics of the conductive filament.

## 1. Introduction

The study of wave propagation in different guiding topologies is a subject that has been extensively treated on the last years. In particular, and on the search for new and higher performance high-frequency devices, many studies on metasurfaces and its applications have been conducted [1,2]. These structures can synthesize different wave behaviours that are not present in nature, like left handed materials, which can have either negative values of permittivity ε or permeability μ [3]. These metasurfaces can be constructed using periodic structures in arrangements of sub-wavelength unit cells, whose dimensions with respect to the wavelength will determine the characteristics of the material [4]. In brief, depending on the dimensions of the structure, the overall behaviour of the metasurface can have either ε or μ negative, or a double negative material, where both ε and μ are negative [5].

Metasurfaces properties have been largely studied for their use in electromagnetic high frequency applications [6,7,8,9]. One interesting application of metasurfaces is the creation of stop-bands, where in a determined frequency band, there is no wave propagation on the structure. This can be achieved using bed of metallic pins [10], metallic spring-like structures [11], or a metallic “mushroom-like” structure proposed in [12], amongst others [13]. The use of metasurfaces to create stop-bans has led to several applications in electromagnetic high frequency topologies, such as electromagnetic packaging to suppress higher order modes in printed circuit boards (PCBs) and amplifiers cavities [11,14,15,16,17], or gap-waveguides [10], which are structures that are widely study, and nowadays candidate for many high frequency applications [18,19,20]. One of the drawbacks of the use of these structures is with respect to its manufacture process. The fabrication of metasurfaces can be complex especially when the periodic structure is not a simple pin or patch. Methods like drilling, milling, or PCB printing are limited to make simple structures where you can only remove material from the top side. This is a problem when you have structures like springs or “mushroom-like” where you need to remove material under other metal surfaces if you do them without a substrate or, when manufactured with a PCB, grounded vias are required which can be expensive. In addition, these structures are usually made of metal, and when increasing the operation frequency, the dimensions of the structure (height and periodicity), can involve a high cost on the fabrication of prototypes when using a traditional manufacturing processes, such as milling. Therefore, the use of 3D-printing is a promising candidate in order to implement these structures and to reduce its fabrication time and reduce the cost of the prototypes [21,22].

Nowadays, 3D-printing has a huge influence on the methods of manufacturing and prototyping. New generation of low-cost printers has improve in terms of the cost-accuracy relation, enlarging the possibilities of manufacture, of structures and models, that were either expensive or difficult to build [23,24]. In addition, the wide range of materials available that can be used for 3D-printing makes possible the manufacture of electromagnetic high frequency structures [25,26]. In terms of cost-precision relation, the additive manufacturing 3D-printers that use filaments are great candidates for fast and low cost prototyping, in comparison with other manufacturing techniques such as stereolithography or laser-based printing. In previous works, various additive manufacturing techniques have been implemented for the fabrication of high-frequency devices, such as antennas [27,28], having good results in terms of matching and radiation characteristics. Nevertheless, there are few studies that implement direct 3D-printing using the conductive filaments available on the market for this purpose [29,30,31,32]. These filaments [33,34,35] are good candidates to implement complex structures such as metasurfaces, under the scope of a low-cost prototyping [22].

One common kind of metasurface is the Sievenpiper “mushroom-like” structure [12]. This kind of metasurface, composed by a top metallic patch with a grounding via to a ground plane, has as main difficulty to implement the via by an easy or cheap method in prototyping. Therefore, this work will focus on the use of a conductive filament named electrifi [33], that can be printed directly with a low-cost 3D-printer without any post-printing treatment, to build a Sievenpiper “mushroom-like” metasurface. We have chosen this kind of metasurface due to its inherent manufacturing difficulties found on their prototyping process. The structure will be fully 3D-printed to compare its performance in terms of losses and stop-band generated, with a simulated ideal case. The article is divided as follows: in Section 2 the design of the 3D-printed metasurface is presented, in Section 3 a parametric simulation study for the candidate metasurface is made, and finally Section 4 contains the manufacture process and measurement results of the metasurface.

## 2. Metasurface Structure Design

Before the implementation of the metasurface using 3D-printing, it is necessary to introduce its key parameters and the manufacture limits that we have in order to design an operational structure that can be manufactured with our available equipment.

### 2.1. Sievenpiper “Mushroom-Like” Metasurface

**Figure 1:**
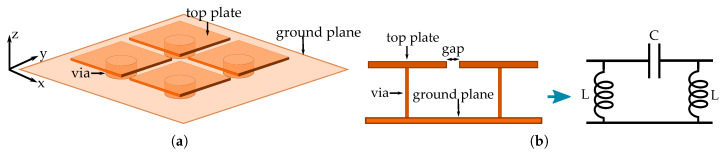
Sievenpiper mushroom-like metasurface. (**a**) Structure and components. (**b**) Equivalent circuit.

A Sievenpiper mushroom-like structure consists of a flat metal top patch that is connected to a conductive ground plane through a metallic via [12]. When this structure is placed into a periodic arrange, the separation between two adjacent mushrooms top patches creates a capacitance, while the conductive via that is connected to the metallic ground plane generates an inductance [12]. This circuital equivalence of these two constructive elements of the mushroom generates a resonance frequency, inducing a high impedance surface that avoids the propagation of surface waves in a frequency range called band-gap or stop-band. Figure 1 shows a description of the mushroom-like Sievenpiper metasurface and its equivalent circuit of two adjacent mushrooms.

### 2.2. Construction Considerations on the Design

**Figure 2:**
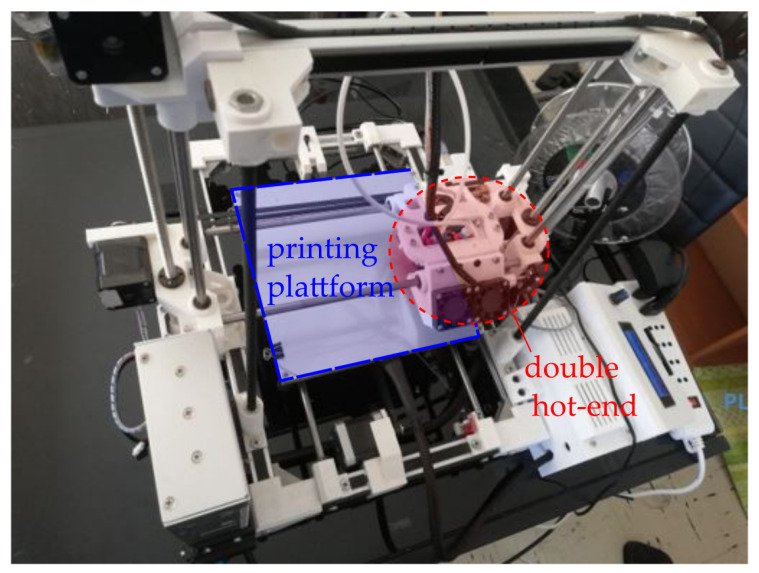
EIE-custom 3D-printer by Ocular3D [36].

One important aspect before doing the design of the mushroom Sievenpiper metasurface is to assess the limitations of the available manufacturing equipment, in order to design a metasurface structure that can be easily constructed with our 3D-printer. The available 3D-printer for manufacture is a EIE-custom 3D printer by Ocular3D [36]. A summary of the most relevant technical characteristics are summarised in Table 1, and Figure 2 shows the EIE-custom 3D-printer available for fabrication.

**Figure 3:**
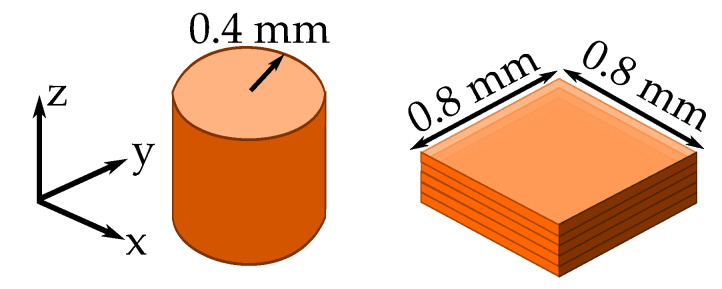
Minimum xy-axis resolution using the conductive filament with the available 3D-printer for different volumetric surfaces: circles and squares.

However, the main limitation to print the structure is not coming from the printer resolution, but from the conductive filament characteristics. The conductive filament that will be used is the electrifi filament, which has the lower resistivity, 0.006 Ω·cm, available on the market for this kind of filaments [33]. The first limitation comes from the resolution that can be obtained using the conductive filament. The design must have a recommended minimum *z*-axis resolution of 0.2 mm due to height stability and expansion of the filament during printing. Higher layer heights can be printed with this filament, but experimentally we achieved better surface finish using the lower range of layer heights (around 0.2 mm). On the other hand, the xy-axis resolution is determined by the nozzle and the conductive filament characteristics. The expansion of the filament and the nozzle dimensions give as a xy-axis single line resolution 0.4 mm. However, this resolution cannot be used to print volumetric structures due to the movement of the nozzle. That is to say, to make structures that can have a z-axis component, the minimum printing surface is a circle with 0.4 mm of radius, or a square with 0.8 mm × 0.8 mm of lateral dimensions (Figure 3). This is because the printer needs to add two minimum resolution lines in order to build a volume in the *z*-axis. This is an important parameter due to the characteristics of the metasurface that we are designing. The conductive via dimensions will be therefore limited to the minimum printed values that can be achieved with the filament.

One final parameter to consider in order to print with this filament is the bridging stability on the printing process. Due to the printing temperature of this material (around 160 ${}^{\circ }$C) it is difficult to print bridged or hanging structures without the use of supports. The use of support structures can have an impact first on the larger amount of conductive filament that will be used, and second, on the printing precision, due to the post-printing removal process. Therefore, this can be a problem to print the via with the top metallic plate of the metasurface. To ease our design and printing process, and due to the fact that the printer has two hot-ends that can be used in parallel, the metasurface will be printed inserted into a polylactic acid (PLA) substrate. The PLA that will be used is the same that was used in [29] from Sakata 3D [38], that was used as a substrate and characterized up to 10 GHz. In addition, the printer can use both materials in each layer, which allows to print the whole metasurface structure without any user intervention during the printing process (i.e., no filament change, nor printing program change).

## 3. High-Frequency Simulation Results

### 3.1. Unit Cell Design

Once the lower limits in terms of fabrication have been determined, we can design a mushroom metasurface taking into account those limitations. One typical analysis that is used to design these periodic structures is to estimate the dispersion diagram of a unit cell of the metasurface by using the irreducible Brillouin zone analysis [39,40,41]. This analysis consists of reducing the periodic structure into a periodic unit cell and calculate the wave vector for each propagating mode, as function of the frequency, on different wave propagation directions for a given number of propagating modes. Therefore, the obtained dispersion diagram will give us information on how the wave propagates through the periodic structure, and if there is any stop-band generated in a frequency range.

**Figure 4:**
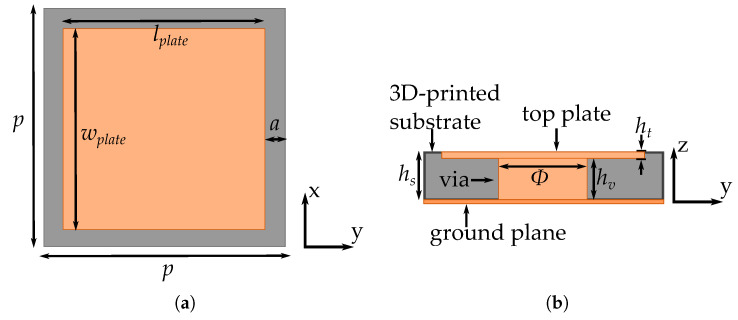
Metasurface unit cell. (**a**) Top-view. (**b**) Cut-view.

The designed unit cell for the Sievenpiper mushroom metasurface is shown in Figure 4. The top plate dimensions are $w_{plate}=l_{plate}$ = 6 mm, while the periodicity of the structure is p= 7 mm, whith a= 1 mm. The substrate used is a 3D-printed Sakata filament with a relative permittivity of $\varepsilon_{r}=$ 2.5 [29], and a height of $h_{s}=$ 1.4 mm. The top plate is inserted with a $h_{t}=$ 0.2 mm due to the aforementioned printing limitations. The conductive via has a height of $h_{v}=$ 1.2 mm with a diameter of Φ= 2.6 mm. For the unit cell analysis, all the materials have to be consider lossless. This means that all conductors are modelled as perfect electric conductors (PEC) and the substrate without dielectric losses. This unit cell will be evaluated for the different cases of use: inside a parallel plate structure and with a suspended microstrip structure.

### 3.2. Metasurface Inside a Parallel Plate Simulation

**Figure 5:**
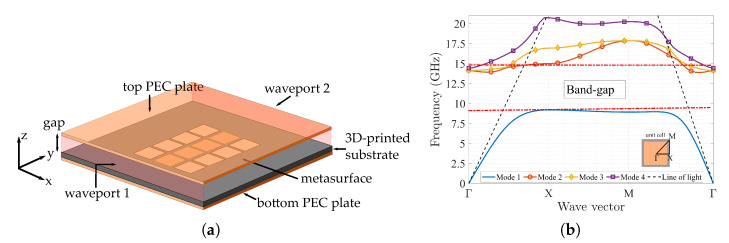
Metasurface inserted in a parallel plate waveguide. (**a**) Structure and components. (**b**) Dispersion diagram of the unit cell.

The first simulation experiment is to place the designed metasurface into a parallel plate waveguide structure. This structure consists of two perfect conductor parallel plates where the metasurface is inserted, having a gap of 1.75 mm between the metasurface and the top metal lid. The dimensions of the mushroom structure are the same used in Figure 4. Figure 5 shows the parallel plate structure with the designed metasurface and the corresponding dispersion diagram. To obtain the dispersion diagrams, the full-wave simulation software CST Microwave Studio [42] is used. The result shows the wave vector of the first four propagation modes in the unit cell, as a function of the frequency and the propagation direction (Γ to *X*, *X* to *M* and *M* to Γ). The wave will propagate on the frequencies outside the stop-band generated by the metasurface, and will be attenuated on the metasurface stop-band. In this case, the stop-band or band-gap is generated between the first and second propagating mode between 9.7 and 14.8 GHz. To notice that, by inserting the periodic structure into the parallel plate structure, we have changed the overall unit cell, by adding a top metal lid over the metal plate of the mushroom, separated by an air gap.

In order to assess the effects of the materials that will be used for the construction of the metasurface, such as the conductive filament finite conductivity and the 3D-printed substrate losses it is necessary co carry a full-wave simulation that can take into account these properties. One way to assess these metasurfaces and verify the produced stop-band is to place a suspended microstrip transmission line over the metasurface and evaluate its transmission coefficient of the scattering parameters of the line [43], or use a parallel plate waveguide and insert the metasurface inside it to also assess the transmission parameters [12]. In other terms, assess the ratio of transmitted signal with respect to the input signal over a large frequency band. If the metasurface is designed correctly, we expect to see an important drop in the transmission parameter at the stop-band frequencies, and high transmission levels outside the stop-band. The effects of the material characteristics can have an influence either on the transmission losses outside the stop-band or in stop-band frequency characteristics. For all the simulations, the full-wave CST software is used.

**Figure 6:**
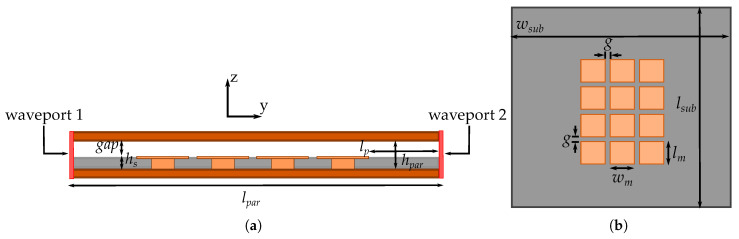
Simulated parallel plate structure. (**a**) Side-view. (**b**) Top-view of the metasurface.

For the full-wave simulation to assess the transmission coefficient we use a 3×4 mushroom periodic structure with the dimensions shown in Figure 6. The larger row of mushrooms is placed alongside the waveport direction of propagation. The parallel plates are separated with a gap of $h_{par}=$ 3.15 mm, while the waveports are separated along the y−axis with $l_{par}=$ 50 mm. The substrate that holds the metasurface has as lateral dimensions $w_{sub}=l_{sub}=$ 50 mm. The mushroom lateral dimensions are $w_{m}=l_{m}$ = 6 mm (same as the unit cell), while the separation *g* between the adjacent mushroom is of 1 mm. The via height, via diameter, and the top-plate height are the used for the unit cell ($h_{v}=$ 1.2 mm, Φ= 2.6 mm, and $h_{t}=$ 0.2 mm respectively). Finally, the metasurface is separated from the waveports with a distance $l_{p}=$ 11.5 mm and from the top PEC plate with a gap of 1.75 mm.

For the simulations, we use different properties of the conductive materials in order to assess their effect over the behaviour of the mushroom structure, while the dielectric remains unchanged. The substrate used for all the simulations is the same 3D-printed substrate used for the unit cell ($\varepsilon_{r}$ = 2.5), but this time we added the estimated dielectric losses tanδ= 0.02 [29]. For the conductive parts of the metasurface, we have chosen four case analysis. First, a full PEC metasurface to be used as the reference case. Second, using the conductivity given by the manufacturer of the electrifi filament, that is around $\sigma =16\times 10^{3}$ S/m. A third case scenario, using a lower conductivity value of σ= 750 S/m and a fourth case that uses PEC for the top plate and for the ground plane, while the via uses the low conductivity value of σ= 750 S/m. This last case was chosen in terms of the possibility of making the structure with a conductive filament, but adding to the top and bottom layers an electrodeposition process post printing, that has been demonstrated to have excellent results with this filament [44].

**Figure 7:**
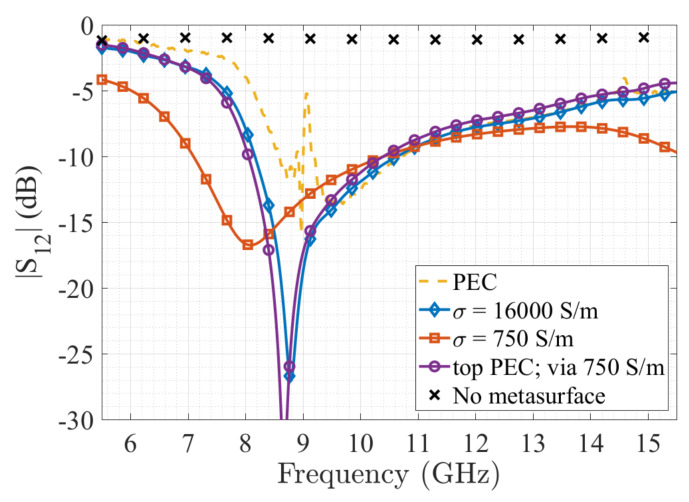
Simulated transmission coefficient $|S_{12}|$ as a function of the frequency of the parallel plate with a 3 × 4 mushroom metasurface, using different conductive materials for the metasurface.

Simulation results (Figure 7 show a good agreement with the band-gap obtained in the unit cell analysis. We can see that the transmission attenuation starts earlier in frequency (around 7.8 GHz), this can be explained due to the more realistic analysis in the full wave simulation [45,46], taking also into account that the metasurface did not cover the whole surface of the parallel plate and that in this simulation we are taking into account the non-ideal characteristics of the materials (i.e., dielectric losses, finite conductivity), and also the reduced number of unit cells used. Nevertheless, we can see the large stop-band obtained with the metasurface. Regarding the analysis of the different conductivity values, we can observe that the ideal PEC case (no conductive losses) has a similar behaviour compared to the $\sigma =16\times 10^{3}$ S/m and the top PEC and via 750 S/m case. This means that the conductivity value of the via is a parameter that can be compensated using high conductivity values on the top and bottom of the metasurface, making it suitable for example for electrodeposition processes that cannot be applied to the inside elements as vias. On the other hand, by reducing the conductivity of the whole metasurface structure (σ= 750 S/m), we can see that there is a slight shift on the stop-band, but mainly we can observe higher levels of attenuation over the transmission coefficient on the whole band.

The parallel plate simulation is a good experiment to verify the metasurface behaviour. Nevertheless, it is hard to implement due to the way of exciting the structure. A common way to measure this structure is to put two radiating elements (e.g., monopoles) in the place of the waveport used in simulations [12]. However, this incurs in an increase on the losses due to the open characteristic of the structure, making harder to analyse the effects of the materials inside of the structure. For this reason, another structure more suitable for construction and measurement will be analysed in full-wave simulation. This structure is a suspended microstrip line which integrates the designed metasurface.

### 3.3. Metasurface with a Suspended Microstrip Line

**Figure 8:**
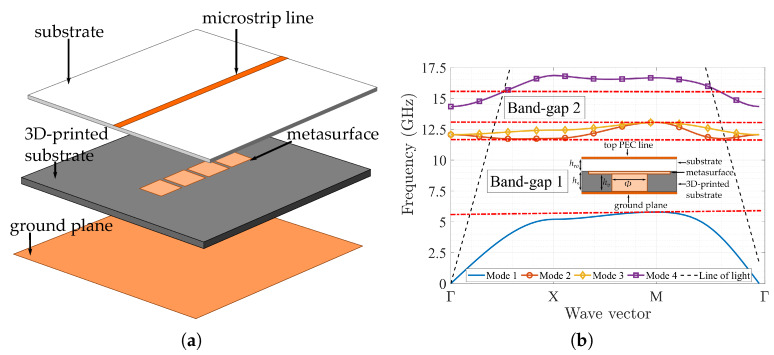
Suspended microstrip with metasurface. (**a**) Exploded view. (**b**) Dispersion diagram with unit cell (cut view).

The suspended microstrip line structure used to assess the performance of a mushroom-like metasurface is a common and easy experiment to manufacture [43]. The device consists of a 50 Ω microstrip transmission line etched on a dielectric substrate, while between the ground plane and the bottom of the dielectric substrate is inserted the metasurface, placing it under the propagation wave path generated between the line and the ground plane. Figure 8a shows an exploded view of this structure.

By placing a substrate over the metasurface, we also change the behaviour of the unit cell with this new dielectric placed over the top plate of the mushroom. Therefore, it is important to calculate the new dispersion diagram generated by this new configuration. The substrate used is a Roger RO4003 ($\varepsilon_{r}=$ 3.55) with a height of $h_{ro}$ = 0.8 mm. The other dimensions of the unit cell remains the same as the case presented in Figure 4. The new dispersion diagram with a cut view of the new unit cell is shown in Figure 8b. As expected, the dispersion diagram changes with respect to the one without the top substrate. We can see the appearance of two band-gaps withing the first four propagating modes. The first band-gap occurs between the first and second mode, from 5.7 to 11.7 GHz, and a second stop band is observed between the third and four mode, from 13 to 15.5 GHz.

**Figure 9:**
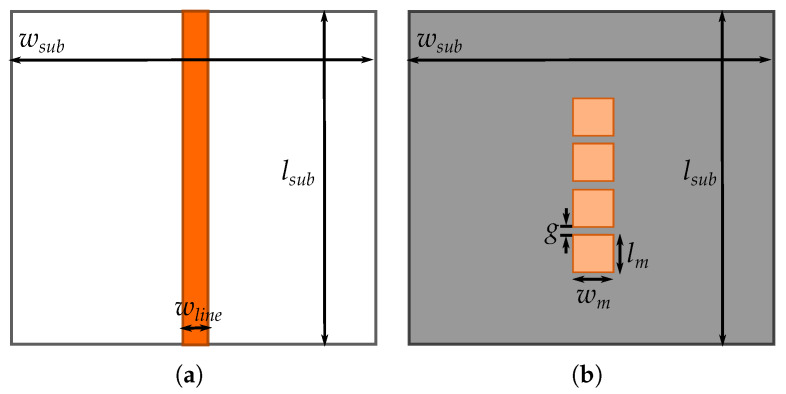
Top view of the designed structures for the suspended microstrip metasurface assessment. (**a**) Top-view of the microstrip line etched on a Rogers4003 substrate. (**b**) Top-view of the 1 × 4 mushroom metasurface.

After the unit cell analysis, we prepare the full-wave simulation using CST. First, and due to the behaviour of the propagating wave along the microstrip line (i.e., most of the field is propagating between the top line and the ground plane), for these simulations we use two different metasurfaces. One metasurface is the same 3 × 4 mushroom metasurface used in the parallel plate simulations, with the same dimensions, and the second metasurface is a 1 × 4 mushroom metasurface, with the same unit cell dimension as the previous one. This assessment is to compare their performance on generating the band-gap and evaluate if due to the field configuration, it is suitable to construct less mushrooms. Figure 9 shows the top view of the microstrip line and the new 1 × 4 metasurface structure. The dimensions of the structures are: $w_{sub}=l_{sub}=$ 50 mm, $w_{line}=$ 1.64 mm, $w_{m}=l_{m}=$ 6 mm, and g= 1 mm.

**Figure 10:**
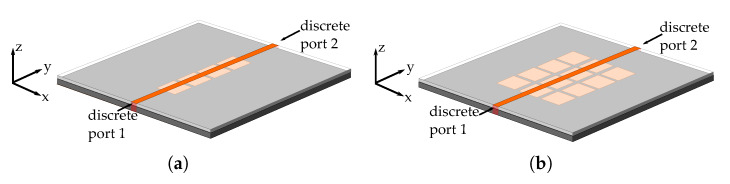
Suspended microstrip 3D design for simulations with different metasurface configurations; (**a**) 1 × 4 mushroom metasurface; (**b**) 3 × 4 mushroom metasurface.

The simulated structures are shown in Figure 10. In order to get closer to a scenario where real SMA connectors are used, the feeding ports are simulated using discrete ports instead of the waveports that were previously used on the parallel plate scenario. The 50Ω microstrip line is etched over a Rogers RO4003 substrate ($\varepsilon_{r}=$ 3.55, tanδ= 0.002), and placed over the 1 × 4 and 3 × 4 mushroom metasurfaces, with a distance to the discrete port of 11.5 mm. The metasurface is integrated in the same Sakata 3D-printed substrate used in the parallel plate ($\varepsilon_{r}=$ 2.5, tanδ= 0.02), and using the same four conductive material cases for the mushroom (i.e., PEC, $\sigma =16\times 10^{3}$ S/m, σ= 750 S/m and PEC plates with σ= 750 S/m via). In addition, all cases are compared to a case using the same structure, but without the mushroom metasurface inserted on the 3D-printed substrate.

**Figure 11:**
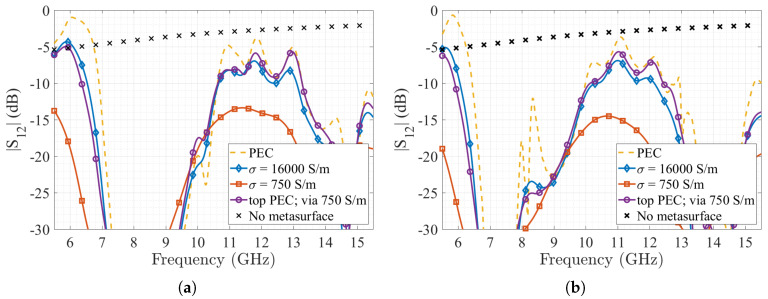
Suspended microstrip transmission coefficient $|S_{12}|$ as a function of the frequency, using different conductive materials for the metasurface; (**a**) 1 × 4 mushroom metasurface; (**b**) 3 × 4 mushrooms metasurface.

The simulation results of the $|S_{12}|$ transmission coefficient as a function of the frequency, for the four different conductive materials and both metasurfaces are shown in Figure 11. We can see that there is a good agreement between the unit cell calculation of the generated stop-bands, and the transmission coefficient attenuation produced by the two stop-bands (i.e., from 5.7 to 11.7 GHz, and from 13 to 15.5 GHz). This is valid for both metasurfaces. Finally, the conductive material behaviour of the metasurface has the same results presented on the parallel plate simulations, in terms of losses and generated stop-band.

## 4. Fabrication and Measurement Results

Once the metasurface is designed and assessed in simulation we have to prepare the 3D-printing process for the materials and finally measure the resulting structures.

### 4.1. Metasurface Fabrication

**Figure 12:**
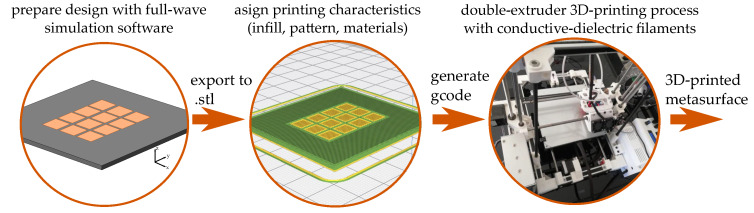
3D-printing process: from design to construction.

In order to print the designed metasurface structure we need to prepare the design to make it suitable for the 3D-printer. The printing process is depicted in Figure 12. First, we use a full-wave simulation software to design the metasurface and export the designed structure into a format (for example .stl), that a conventional 3D-printing software handler can process (e.g., Ultimaker CURA [47]). Once the design is uploaded into the 3D-printing software, all the printing parameters are set. In this step, the materials, infill percentages, infill pattern, temperatures and printing speeds are set for each extruder, depending on the filament that is loaded into it. The last step is the generation of the gcode to print the device using the double extruder 3D-printer.

For this study, the design of the metasurface was done using CST Microwave Studio. Each part of the structure (substrate, mushroom and ground plane) needs to be exported separately. The 3D designs were imported to Ultimaker CURA, and joint them into one printing file. During this step, the 3D-printer profile and materials profile are uploaded. The materials to be used in the printing are the electrifi conductive filament for all the conductive parts of the metasurface (mushroom and ground plane), and the Sakata PLA filament for the dielectric substrate of the metasurface. Due to the physical characteristics of the conductive filament, it has to be printed using temperatures around 160 ${}^{\circ }$C and a printing speed below 15 mm/s to avoid the rupture of the filament. To notice that as this printing process uses a double extrusion, the leveling in calibration is crucial to avoid the destruction of the pieces, due to the different printing temperatures on each hot-end. Table 2 shows the printing parameters used for the additive manufacturing of the metasurface.

**Figure 13:**
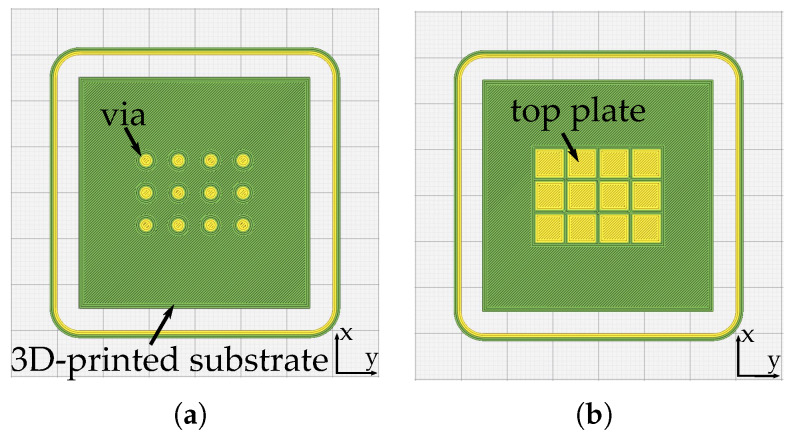
Top view of CURA print preview. (**a**) Middle layer. (**b**) Top layer.

The advantage of the use of double extrusion for this printing process is that as the 3D-printing is done in layers, each layer material is printed at the same time on each layer. That is to say, the substrate and the vias are printed at the same time, which helps the conductive filament to fit perfectly into the cavity that is creating the PLA substrate, ensuring the correct dimensions and shapes of the metasurface. Figure 13 shows the middle and top layers of the printing process.

**Figure 14:**
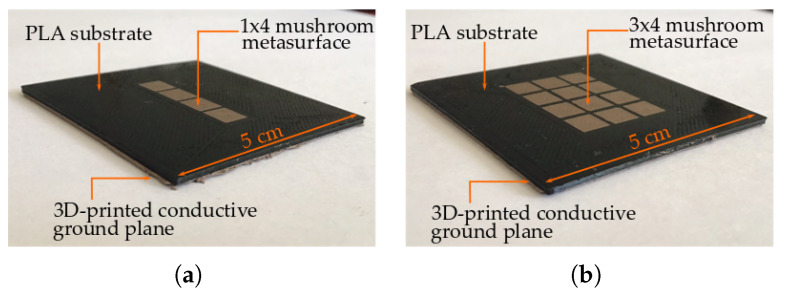
3D-printed metasurfaces. (**a**) 1×4 mushroom with printed conductive ground plane. (**b**) 3×4 mu shroom with printed conductive ground plane.

The constructed devices are shown in Figure 14. For the experiments, the 1×4 mushroom and the 3×4 mushroom metasurface were build with a 3D-printed conductive ground plane.

### 4.2. Measurement Results

**Figure 15:**
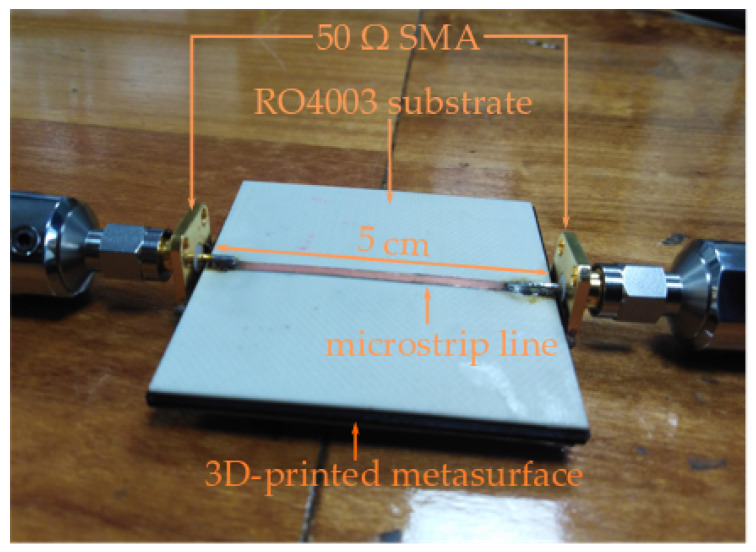
Constructed metasurface with suspended microstrip line.

**Figure 16:**
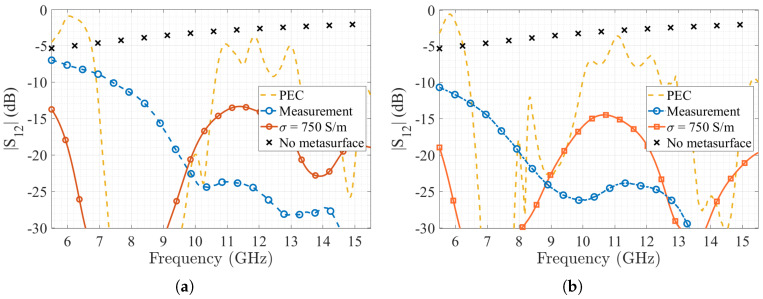
Measurement results of the transmission coefficient as function of the frequency and compared with simulated results for the two constructed metasurfaces with a suspended microstrip line; (**a**) 1×4 mushroom metasurface; (**b**) 3×4 mushroom metasurface.

For the measurement of the metasurface, the suspended microstrip line method was chosen. A 50 Ω microstrip line was etched using a Roger RO4003 ($\varepsilon_{r}=$ 3.55) with a height of $h_{ro}=$ 0.8 mm. The substrate is placed over the metasurface and two 50 Ω SMA connectors are soldered to the line and the ground plane. For the measurement of the transmission coefficient, a Vector Network Analyzer ANRITSU MS46122B was used. The measured structure is shown in Figure 15 and its transmission coefficient as a function of the frequency, for the cases 1×4 and 3×4 metasurfaces, is shown in Figure 16.

The measurements obtained are compared with two of the previously simulated cases, i.e., the simulated PEC case for the 1×4 and 3×4 metasurfaces, the same structures using a conductivity of 750 S/m, and with the reference case when no metasurface is added to the 3D-printed substrate. We can see that both stop-bands are present on both measurement results, with the first band starting at 5.7 GHz up to 11 GHz, and the second stop band starting on 13 GHz, which agrees with the frequencies obtained in simulations. However, losses are higher than the simulated ones, mainly on the transmission band between 11.7 and 13 GHz, with results closer to the low conductivity simulation. This can be explained due to the conductive filament losses presented on the mushroom and the fully printed conductive ground plane. These losses were measured in [29], and are around 0.12 dB/mm at 10 GHz and correspond to the expected values according to the dimensions of the 3D-printed structures. As stated before, the losses can be compensated by using a superficial one-step copper electrodeposition, as was demonstrated in [44] with excellent results in terms of obtained conductivity using this same filament, even on its use with radiating elements at 30 GHz.

## 5. Conclusions

This article presents the design, construction and measurement of two mushroom metasurfaces using 3D-printing with conductive PLA filaments for the metallic parts. Measurement results of the 3D-printed metasurfaces show the appearance of the stop-bands at the simulated expected frequencies, having slight differences that can be attributed to the materials properties tolerances. However, due to the high losses introduced by the conductive filament, the higher transmission band is severely attenuated in comparison with the ideal PEC case. This is in agreement with other publications that characterize this filament in high electromagnetic frequencies. Unfortunately, there are no better conductive filaments in the market that are compatible with low cost 3D-printing and compatible with the simultaneous use with other standard filaments (i.e., no additional post-processing like sintering needed). However, these losses can be easily compensated by adding an electrodeposition process to the top and bottom of the metasurface structure, which is a process that was demonstrated in other publications that is suitable for this particular conductive PLA. In addition, it was demonstrated through simulation that the via conductivity is not the main parameter for a correct performance of the metasurface in terms of transmission losses, which implies that the via does not need an improvement of its conductivity, so the manufacturing process remains an easy and low-cost process.

## Figures and Tables

**Table 1:** Ocular3D EIE-custom 3D printer technical specifications.

Printer Parameter	Value
Maximum printing volume	190 × 190 × 190 mm${}^{3}$
Axis resolution	100 μm in all axis *(xyz)*
Hot-end model	Two E3DV6 [37]
Hot-end T${}^{\circ }$ range	120 to 280 ${}^{\circ }$C
Platform maximum T${}^{\circ }$	100 ${}^{\circ }$C

**Table 2:** 3D-printer settings for conductive filament.

Printing Setting	Extruder 1	Extruder 2
Layer height	0.2 mm	0.2 mm
Wall Thickness	0.4 mm	0.8 mm
Infill percentage	100%	100%
Infill pattern	lines	lines
Printing temperature	160 ${}^{\circ }$C	210 ${}^{\circ }$C
Stand-by temperature	145 ${}^{\circ }$C	190 ${}^{\circ }$C
Printing speed	15 mm/s	40 mm/s
Z hop when retracted	Yes	Yes
Cooling fan	100%	100%
Flow	120%	100%
